# Optogenetic Stimulation Enhanced Neuronal Plasticities in Motor Recovery after Ischemic Stroke

**DOI:** 10.1155/2019/5271573

**Published:** 2019-03-14

**Authors:** Changbo Lu, Xianglong Wu, Hongzhe Ma, Qingchuan Wang, Yikai Wang, Yan Luo, Cong Li, Hui Xu

**Affiliations:** ^1^Department of Neurobiology and Collaborative Innovation Center for Brain Science, School of Basic Medicine, Fourth Military Medical University, Xi'an 710032, China; ^2^Key Laboratory for Space Bioscience and Biotechnology, School of Life Sciences, Northwestern Polytechnical University, Xi'an 710072, China; ^3^Department of Anesthesiology, Heze Municipal Hospital, Clinical College of Taishan Medical University, Heze 274031, Shandong, China

## Abstract

Motor capability recovery after ischemic stroke involves dynamic remodeling processes of neural connectomes in the nervous system. Various neuromodulatory strategies combining direct stimulating interventions with behavioral trainings for motor recovery after ischemic stroke have been developed. However, the effectiveness of these interventions varies widely due to unspecific activation or inhibition of undefined neuronal subtypes. Optogenetics is a functional and structural connection-based approach that can selectively activate or inhibit specific subtype neurons with a higher precision, and it has been widely applied to build up neuronal plasticities of the nervous system, which shows a great potential in restoring motor functions in stroke animal models. Here, we reviewed neurobiological mechanisms of enhanced brain plasticities underlying motor recovery through the optogenetic stimulation after ischemic stroke. Several brain sites and neural circuits that have been previously proven effective for motor function rehabilitation were identified, which would be helpful for a more schematic understanding of effective neuronal connectomes in the motor function recovery after ischemic stroke.

## 1. Introduction

Ischemic stroke, a leading cause of severe disabilities in the adult population, has an annual economic cost of approximately $34 billion all over the world [1]; it also brings about a large number of familial and social burdens in lost productivity. A stroke occurs when a loss of blood flow disables both afferent and efferent connectivities, which finally result in a loss of sensory and motor connections to the world. A number of neurological functions are impaired after a stroke, among which motor disability contralateral to the stroke lesion side accounts for most dysfunctions [2]. Autonomously, the brain functional networks undergo the reorganization and rewiring after a structural lesion from a stroke [3–5]. These remodeling processes are often associated with the sprouting of spared axons that can innervate denervated target areas and the establishing of new circuits for the recovery of lost functions [6–8]. Such spontaneous reorganizations of connectomes can partially restore motor functions, but the effects are limited and can sometimes be harmful to neurological recovery in large stroke lesions [9]. To maximally regain sensory and motor functions after stroke, surviving neural circuits should be regularly reorganized to make new connections [3, 10, 11]. Thus, multiple strategies have been introduced to promote better neural rewiring and ultimately achieve better recovery of the motor function [11]. These strategies include pharmacological treatment, rehabilitation, cell transplantation and brain stimulation [12–16]. Among them, brain stimulation is considered as a direct and promising neurorestorative strategy. For one thing, neuronal activity is a major factor that influences neural plasticity and the recovery from ischemic stroke [3, 17]. For another reason, the brain stimulation allows direct manipulation of the activities of a specific brain area, as evoked cortical activities in the damaged hemisphere are decreased after focal stroke [5, 7, 8, 18]. Traditional brain stimulation strategies include microelectrode stimulation, deep brain stimulation, and noninvasive stimulation methods, such as transcranial magnetic stimulation and transcranial direct current stimulation (tDCS). All these stimulations have been shown to promote motor function recovery [19–21]. However, an intrinsic limitation of these approaches is that the cellular effects of TMS or tDCS are much less focal: both of them activate mixed populations of neurons (regardless of neurons or astrocytes), thus blurring the underlying mechanisms of stimulation and producing a range of effects [22–24]. Consequently, which brain areas or circuits are activated or inhibited, and whether stimulations of these targets are efficacious in terms of the motor functional recovery, are still poorly known so far. With the emergence of the optogenetic approach, specific cell types and neural circuits could be well activated or inhibited with a high precision both temporally and spatially [25–27]. For instance, one study comparing optogenetic versus electrical stimulation of dopamine terminals in the nucleus accumbens showed that electrical stimulation induces both local multisynaptic and indirect modulation of DA release, which are absent in optogenetically targeted stimulation [28]. Here, we reviewed recent studies on optogenetic techniques aiming at rewiring or reestablishing brain circuits for motor function regain in stroke rodents. We have summarized several effective target circuits that have the potential for neuromodulatory intervention in the brain after stroke. These potential targets include the ipsilesional motor cortex (iM1), the afferent circuits to the motor area, the efferent corticospinal circuits, and the neurogenic niche subventricular zone (SVZ) (Table 1). Notably, the viral vectors with promoters such as CaMKII to drive the expression of ChR2 in neurons of target regions will allow for selective stimulation of certain subtype neurons, which is critical in understanding the ultimate neuronal activity in functional restoration after stroke. More importantly, the underlying neurobiological mechanisms on mesoscopic (regional interactions) and microscopic (synapses) levels are also elucidated, which might provide an advantage for other stimulation methods, possibly by offering similar or greater therapeutic efficacy in clinical works.

## 2. Mesoscopic Plasticity of Brain Sites and Circuits in the Motor Restoration after Ischemic Stroke

### 2.1. Enhanced Neuronal Activities in Ischemic Infarcted iM1 and Peri-Infarcted Areas

In normal conditions, each cortical hemisphere inhibits the other through transcallosal fibers [29]. Stroke disrupts the interinhabitation mechanism and results in excessive inhibition from the contralesional cortex, where evoked cortical activity decreases [30]. Within the ipsilesional cortex, a significant increase in tonic neuronal inhibition in the peri-infarct zone has been detected after stroke [5]. Based on this interhemispheric competition model and tonic inhibition mechanisms in the peri-infarct zone, the iM1 of the damaged hemisphere became one of the target cortices for the stimulation. A preclinical study revealed that invasive cortical electrical stimulations in the ipsilesional M1 enhanced dendritic plasticities in both the peri-infarct areas and contralateral cortex after stroke [21]. However, limited by indiscriminate activation of all cell types near the stimulated site, the actual effects and mechanisms driving motor recovery remained uncertain. The first attempt using optogenetics to manipulate specific cell types to promote stroke recovery was studied [31]. Optogenetics was used to excite neuronal activities selectively in the ipsilesional primary motor cortex (iM1) poststroke in transgenic mice expressing ChR2. During stimulation, the laser was set to 10 Hz, 20 ms light pulses with a power range of 0.4–0.8 mW. Recordings in the ipsilesional striatum (iStr) and ipsilesional somatosensory cortex (iS1) revealed that iM1 neuronal stimulation could activate peri-infarct regions (Str and S1) as well as cM1. Moreover, repeated neuronal stimulations within the iM1 significantly promoted corresponding motor function recovery after stroke. Although no direct observation on the structural remolding of axons was made, mechanically, the stimulation promoted the expression of activity-dependent neurotrophins and GAP43, a growth-associated protein critical for axonal sprouting. GAP43 expression was mainly upregulated after stroke in the peri-infarct areas and the contralesional cortex, suggesting that new connections formed from the motor cortex to the premotor cortex and primary and secondary somatosensory cortices [32, 33]. Moreover, these patterns of axonal sprouting overlapped with changes in human functional maps of motor control in peri-infarct motor, premotor, and somatosensory areas [34].

As tonic neuronal inhibition increased in the peri-infarct zone after stroke, to help regain the excitatory-inhibitory balance of the ischemic region within the infarct hemisphere, perhaps direct manufacturing of GABAergic neurons in the peri-infarct zone can become a candidate target for optogenetic stimulation in the future [35, 36].

### 2.2. Reorganization of Afferent Circuits Restored the Motor Function

Although iM1 stimulation has demonstrated significant effects in promoting motor function recovery, the overall motor performance of the mice only recovered to about 50% of the prestroke baseline [31]. To optimize the effect of stimulation-promoted recovery, we focused on the contralesional lateral cerebellar nucleus (cLCN), a deep cerebellar nucleus that sends major excitatory output to multiple motor and sensory areas. Electrical stimulation studies have demonstrated that chronic electrical stimulation of the cLCN could enhance contralateral cortical excitability, which in the end enhances motor function recovery in rats after stroke [37, 38]. With the method of optogenetics, researchers demonstrated that selective stimulation of neurons in the cLCN produced more robust and persistent motor function restoration than the iM1 stimulation. The stimulation laser was set to 10 Hz, 20 msec light pulses with a power range of 0.2–0.4 mW, which mainly stimulate neurons in the LCN with the Thy1 promoter. In addition, repeated cLCN stimulations enhanced the structural plasticity for GAP43 in the ipsilesional somatosensory cortex, suggesting that new connections formed in the joints between afferents from cLCN and the ipsilesional somatosensory cortex [39]. However, as LCN contains three neuronal subtypes including glutamatergic, GABAergic, and glycinergic neurons [40, 41], it calls for more studies to interrogate the effects of stimulating selective neuronal subtypes in the LCN during poststroke recovery.

Apart from afferent cLCN, sensory-evoked cortical activity also depends on the integrity of afferent inputs from the thalamus. It has been shown that focal ischemia leads to a loss of thalamic axonal synaptic contacts with the peri-infarct cortex, resulting in a reduction in the excitability of surviving thalamocortical circuits [42–44], which are proven to be partly responsible for the well-known deficits in superficial (layers 1–3) cortical responses to sensory stimuli after stroke [17, 45]. During the stimulation, the light was set to a 5 Hz, 5 ms light pulse at 10 mW mm^−2^ that reliably activates cortical neurons. As expected, optogenetic stimulation targeting this circuit resulted in a persistent improvement in sensory cortex and paw function. Furthermore, chronic optogenetic stimulation also promotes rewiring of thalamocortical boutons as well as growth and retraction of thalamocortical axon branches [46]. It is worth mentioning that the stimulation focused mainly on thalamocortical sensory circuits rather than those responsible for motor output, providing new cues for mechanisms underlying optogenetic stimulation in improving motor recovery. As the study did not assess the expression of trophic factors in the contralateral hemisphere, the mechanisms underlying the function recovery remain undetermined.

### 2.3. Reorganization of Efferent Circuits Restored the Motor Function

Ischemic stroke disables not only afferent circuits, but also efferent connections. Experimental studies have shown that a stroke denervated corticospinal neurons in the contralateral cortex. Furthermore, after various therapeutic and rehabilitative interventions, the corticospinal tract (CST) on the contralesional side can sprout and terminate in the denervated hemispinal cord [47–49]. Thus, CST has been identified as another critical pathway where rewiring may be associated with the recovery of impaired motor functions after ischemic stroke [50]. Recently, a study by Wahl's group strongly proved the existence of this association. After a large sensorimotor stroke, photoactivation of intact CST neurons in the contralesional motor cortex in combination with motor training can robustly induce CST sprouting of preexisting ipsilaterally projecting axons as well as midline crossing CST fibers in the denervated cervical hemicord [9]. Together with a structural rebuilding of the efferent circuits, light stimulation restored the motor function of the corresponding rats by nearly 100% of their prestroke forelimb movement abilities in the fourth week after the stroke. The stimulation laser was set to a 10 Hz, 20 ms light pulse at 16.6 mW/mm^2^, which specifically activated corticospinal projecting neurons. The encouraging effects of this stimulation paradigm were comparable to previously established anti-Nogo-A immunotherapy [51], verifying the growth-promoting effect induced by optogenetic-based stimulations. Interestingly, the initially reestablished motor function can be extinguished by further optical silencing of newly sprouted fibers of intact CST neurons. Such dual effects confirmed that specific axonal sprouting in contralateral corticospinal circuits is causally associated with recovery in these large-volume stroke models. Furthermore, it is worth noting that the above study incorporated rehabilitative training into the recovery protocol and demonstrated that stimulation-promoted intrinsic circuitry might be significantly strengthened by rehabilitative training through the reshaping of specific circuits. Physical therapy is a critical approach in improving motor function after stroke, and robot-based rehabilitation has been shown to promote network plasticity and functional recovery [52]. Indeed, the formation of a new circuit can be enhanced by growth stimulatory therapies, followed by rehabilitative training and the selective shaping of new circuits for motor functions [53]. However, the neural mechanisms underlying this synergistic effect is still unclear. Accompanied by optogenetics, the combined protocol could allow for an enhanced knowledge of the neural processes underlying the synergistic effects and potentially help to improve the effectiveness of rehabilitative therapy.

## 3. Enhancing Neurogenesis Restored the Motor Function after Ischemic Stroke

Brain stimulation strategies based on activity-dependent neuronal plasticity have made great advancements in motor recovery after stroke. Nevertheless, effective treatment of ischemic stroke remains a major challenge mainly because the narrow treatment window of currently available treatment approaches is limited to the acute phase [54]. Recently, strengthening neurogenesis has become a topic of interest in addressing the narrow treatment window of stroke since it was reported that the brain is capable of generating new neurons [55, 56]. In the adult brain, the subventricular zone (SVZ) of the lateral ventricle and the subgranular zone in the hippocampus are primary neurogenic niches where neural stem cells and neuroblasts inhabit. The striatum is anatomically adjacent to the SVZ and has been proved to project dendrites and axons into the SVZ. Thus, the striatum is presumably in a suitable location to affect cellular activities in the SVZ. Previous studies reported that direct current stimulation of striatal cells in the chronic phase of stroke promoted recovery [57]. Using optogenetics, a past study demonstrated that selectively activating glutamatergic neurons/axons in the striatum triggered a cascade of SVZ cellular responses with increased regenerative activities and functional recovery in the ischemic brain [58]. Furthermore, laser stimulation-induced increases of cells were mainly concentrated in the peri-infarct cortex. Additional behavior tests of the study revealed that regained motor function paralleled with the migration of newborn DCX+ neuroblasts from the SVZ to the peri-infarct region. Thus, the immediate effect of the current stimulation of striatal cells might act through activating glutamatergic neurons/axons in the striatum. Inhibiting rather than exciting the activity of striatal neurons after ischemia improved motor function in stroke mice.

Aside from the stimulation of glutamatergic neurons, GABAergic neurons are known to constitute the majority of striatal neurons [59]. Another study on striatum focused on these neurons. In this study, researchers used a stimulation laser with a 20 Hz, 5 ms light pulse at 1 mW/mm^2^ and a 473 nm-pulse blue laser with 0.5 mW power to specifically inhibit or activate GABAergic neurons in Gad2-Arch-GFP transgenic mice, respectively. It was demonstrated that optogenetic inhibition rather than activation of striatal GABAergic neurons promoted neurogenesis after cerebral ischemia by projecting dendrites and axons to the SVZ [60], leading to a recovery in corresponding motor function in an experimental ischemic stroke model [54].

Altogether, these studies partially illuminate the underlying mechanisms of how altering neuronal activity regulates neurogenesis in a semiphasic synaptic way. This photoactivation of neural regeneration may provide a promising direction in extending the treatment window for stroke.

## 4. Microscopic Mechanisms of Optogenetically Promoted Neuronal Plasticities in the Motor Restoration after Ischemic Stroke

Motor function recovery after stroke depends on the reorganization of neuronal connections throughout the central nervous system (CNS). The contents above have elucidated several potential brain circuits or sites where new connections can grow after optogenetic stimulation. However, the mechanisms of how new functional circuits are formed remain poorly known. We examined three neurobiological mechanisms underlying optogenetic-stimulation-promoted motor function recovery on a synaptic level, including synaptic plasticity, axonal sprouting, and dendritic morphology changes.

Among them, synaptic plasticity refers mainly to the function of synaptic circuits or changes in inhibitory control of circuits. While axonal sprouting and dendritic morphology changes are the structural basis of new connection formations, the axonal sprouting in peri-infarct and connected cortical areas is causally associated with motor recovery and can be triggered by synchronized low frequency neuronal activity [61]. Although different optogenetic stimulation strategies or brain targets lay particular stress on different mechanisms above, neuronal activity is proposed to be the most important driving force in incorporating all these mechanisms of synaptic connections and reorganization of adult cortices [54]. Thus, the internal molecular substrates that underlie these persistent anatomical changes may overlap greatly.

As with activity-dependent synaptic plasticity, both long-term potentiation (LTP) and long-term depression (LTD) have been proposed to be involved in cortical plasticity [62, 63]. It has been proved both in vitro and in vivo that optogenetics can drive synaptic plasticity. Firstly, synaptic composition and function can be directly tuned with light [64]. Secondly, optogenetics can promote selective regeneration of refractory axons in living vertebrates [65]. Indeed, almost all processes of the electrical firing of neurons in the brain are complex assortments of neuronal signaling proteins, including myriad ion channels and receptors. It is well known that the cAMP-dependent pathway is one of the neurobiological basis of optogenetics-promoted synaptic recovery [66]. Diverse forms of synaptic plasticity rely on postsynaptic mechanisms that converge in the AMPA-type glutamate receptors on the postsynaptic membrane. The recruitment of AMPA receptors to PSD by photostimulation has been observed. Once these artificially recruited receptors have access to the synapse, they would occupy the same sites occupied by receptors recruited through synaptic activity [64]. Furthermore, rehabilitation training is helpful in optogenetically stimulated motor recovery after stroke. Specifically, the second messenger cyclic adenosine monophosphate (cAMP) acts as a critical factor promoting axonal regrowth [2–5].

As to axonal sprouting, several sprouting transcriptomes and molecules like EphA4, ATRX, Lingo1, and IGF have been evaluated to be responsible for axonal sprouting after stroke [32, 34, 67]. Among them, EphA4 and Lingo1 are growth inhibitors, while IGF is a growth-associated factor. Activity shapes neural connections by inducing both growth-promoting and growth-inhibiting molecules and neutralizing or enhancing either subset to drive structural and anatomical changes [68]. These paradigms are key signaling pathways for activity-dependent plasticity in the adult brain. However, whether the prompting of new connections by optogenetics shares similar mechanisms with these transcriptome and molecular paradigms is still uncertain.

The dendritic morphology of a neuron determines where and how the new connections of that neuron will be made [69]. Although there is only a finite amount of evidence on optogenetic stimulation-related dendritic morphology changes after stroke, it has been shown that optogenetics can regulate the remodeling of dendritic spines in neurons of patients with Alzheimer's disease by modulating the activity of cofilin, a small protrusion on the surface of dendrites that receives inputs from neighboring neurons [70].

Based on evidence from the microscale synaptic level to the mesoscale circuit level, optogenetics can be utilized as a useful tool to sculpt brain connectivity after stroke. However, since the establishment of connections requires guiding axons to correct target areas and forming synapses at particular regions on postsynaptic neurons, there is still no clear mechanistic rationale on molecular details of the process. Consequently, it calls for more mechanistic studies of neuronal plasticities to advance in developing effective treatment strategies.

## 5. Conclusions and Future Implications

Overall, stroke can be viewed as a disruption of old brain connectomes as well as an origin of new neuronal connection reorganization throughout the central nervous system. Optogenetics is a useful and neuron-specific tool in promoting neural plasticity to benefit motor function rehabilitation after ischemic stroke. Several proved-to-be effective neural circuits have been identified by optogenetics. Among these stimulation targets, recovery through the stimulation of the primary site of ischemia iM1 is mainly involved in potentiating synaptic plasticity within the core of the infarcted area and promoting new connections within peri-infarct areas by enhancing axonal sprouting. Stimulation of afferent and efferent circuits are mainly involved in axonal sprouting within the ipsilesional somatosensory cortex, the thalamocortical circuit, and CST fibers in the denervated cervical hemicord. Optogenetic stimulation of SVZ, which is a niche for the genesis of neuroblasts, involves promoting neuroblasts to migrate to the boundaries of the infarct zone, to differentiate into mature neurons, and to integrate into neuronal networks [71].

Optogenetics-enhanced neural plasticities include both long-term potentiation (LTP) and long-term depression (LTD) [72]. At the synaptic level, optogenetics-enhanced neural plasticity involves recruiting AMPA-type glutamate receptors and increasing the efficiency of synaptic transmission after stroke [64]. Furthermore, optogenetics stimulation can promote axonal sprouting. Although a great many molecules like GDF10, Lingo1, and EphA4 are proven to be critical in the formation of new axonal connections [34], these identified molecules are pleiotropic cytokines or growth factors with effects in the noncentral nervous system. Future therapeutics aiming at enhancing axonal sprouting in stroke models might tap into molecules that are both neural activity-dependent and critical to axonal sprouting in the central nervous system [73]. Nevertheless, all the stimulation targets corresponding to poststroke events are mainly due to the removal of inhibition, activity-dependent synaptic changes, growth of new connections, or unmasking of preexisting connections [74].

Apart from these brain targets, it is worth noting that the contralateral hemisphere has always been of great interest as a target of neuromodulatory interventions [75]. In animal models and human stroke patients, it has been found that increased inhibition in the ipsilesional hemisphere from the hyperexcitable contralesional hemisphere may limit functional recovery after stroke [76, 77]. As traditional rTMS protocols enhancing excitability in the peri-infarct tissue of the ipsilesional hemisphere or downregulating the excitability in the unaffected hemisphere can only grossly rebalance hemispheric excitability, many inconsistencies are generated between studies investigating the effects of rTMS on motor function [78–80]. For these inconsistencies, optogenetic modulation of specific interhemispheric circuits might be a novel approach to specifically modulate the neurons underlying abnormal interhemispheric inhibition after stroke. Due to the imprecise laser power for the stimulation, stimulation parameters like the frequency, duration, and intervals of stimulations should be determined empirically depending on the brain region and cell type of interest [81]. Some stimulation protocols are intended to activate certain neurons, while others are aimed to inhibit target neurons. Depending on the type of opsin used, the stimulation frequency and interval should match the intrinsic photocycle for high stimulation efficiency and low opsin desensitization [24, 82].

Although optogenetics itself could not be applied directly to clinical patients, these studies can be instructive in identifying specific excitatory or inhibitory circuitry that may work in human patients, thus allowing for more precise and effective outcomes for noninvasive and mature stimulation toolboxes like transcranial magnetic stimulation (TMS) or tDCS.

**Figure 1:**
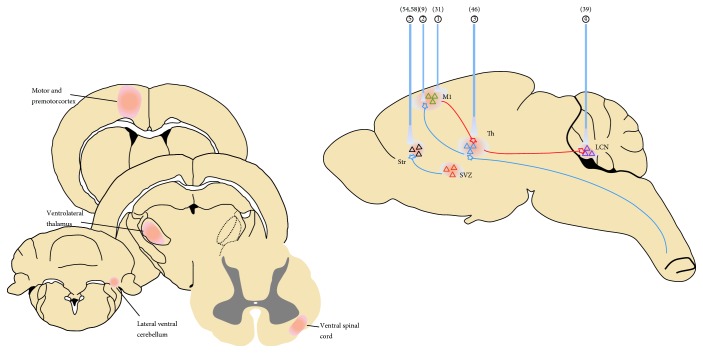
Optogenetic targeting of neural circuits in the motor recovery after ischemic stroke in the mouse brain. The illustration depicts several targeting circuits or sites, (1) including stimulation of ischemic infarcted iM1 [31], (2) stimulation of efferent iM1-thalamus-spinal circuits [9], (3) stimulation of afferent thalamocortical circuits [46], (4) stimulation of multisynaptic projections of cLCN-thalamus-M1 [39], and (5) stimulation of striatum-SVZ projection for neurogenesis [54, 58]. Afferent and efferent circuits are distinguished as blue and red color, respectively.

In conclusion, optogenetics is a useful tool in extending the boundaries of poststroke rehabilitation of motor function by providing a conceptual framework to improve established clinical stimulation techniques (Figure 1). The development of optogenetics has made it evident that brain plasticity is fundamentally a synaptic phenomenon that is largely stimulus dependent, and that brain repair must incorporate biological interventions like brain stimulations and behavioral interventions that are carefully tailored for the reorganization of specific brain circuits [83]. However, more efforts are needed in exploring the basic mechanisms of poststroke paresis as well as the rehabilitation process to guide the tendency in neural repair. Future preclinical trials can mainly focus on two potential directions: firstly, interrogate the effects of stimulating selective neuronal subtypes during poststroke recovery, and secondly, explore how this approach could be integrated with rehabilitation training and biological approaches to take maximum advantage of the brain's capacity to restore its neural networks after stroke.

## Figures and Tables

**Table 1:** Summary of targeted brain circuits or sites in motor recovery after ischemic stroke using the optogenetic stimulation.

Experimental ischemic stroke model	Target method	Stimulation protocol	Stimulation time course	Recovery onset	Ref.
Mice, transient middle cerebral artery occlusion	Thy-1–ChR2–YFP line-18 transgenic mice which exhibited high levels of ChR2 in layer V of primary motor cortex pyramidal neurons	Activated pyramidal neurons in iM1 with 473 nm blue laser	Poststroke days 5-10, 10 Hz	Significant improvement from day 14	[31]
Mice, transient middle cerebral artery occlusion	Thy-1–ChR2–YFP line-18 transgenic mice	Activated neurons (mainly excitatory neurons) in cLCN with 473 nm blue laser	Poststroke days 5–14, 10 Hz	Significant improvement from day 14.	[39]
Rat, middle cerebral artery occlusion	Injection of a Cre-recombinase-dependent ChR2 vector achieved the specific expression of ChR in corticospinal-projecting neurons	Activated corticospinal projecting neurons specifically expressing ChR2 with 473 nm blue LEDs	Poststroke days 3–14, 10 Hz	Significant improvement from day 21	[9]
Mice, ligations of distal branches of the middle cerebral artery	ChR2 transgenic mice (B6.Cg-Tg (Thy1-COP4/EYFP)18Gfng/J) and ChR2-YFP protein were selectively expressed in the excitatory glutamatergic neurons	Activated glutamatergic neuron in the striatum of the ChR2 transgenic mouse with 473 nm blue laser	Poststroke days 5–10, 10 Hz	Significant improvement from day 3	[58]
Mice, middle cerebral artery occlusion	Gad2-Arch-GFP transgenic mice and Arch-GFP were selectively expressed in GABAergic neurons expressed	Inhibited GABAergic neurons in striatum with 530 nm green laser	Poststroke days 5–14, 10 Hz	Significant improvement from day 14	[54]
Mice, middle cerebral artery occlusion	Injection of AAV2.CaMKII.hChR2(E123A) which drove ChR2 expression in thalamocortical projection neurons	Activated thalamocortical projecting neurons with 475 nm blue light	Poststroke days 3-42, 5 Hz	Significant improvement from day 14	[46].

## References

[1] Mozaffarian D., Benjamin E. J., Go A. S. (2015). Heart disease and stroke statistics—2015 update: a report from the American Heart Association. *Circulation*.

[2] Dimyan M. A., Cohen L. G. (2011). Neuroplasticity in the context of motor rehabilitation after stroke. *Nature Reviews Neurology*.

[3] Murphy T. H., Corbett D. (2009). Plasticity during stroke recovery: from synapse to behaviour. *Nature Reviews Neuroscience*.

[4] Ward N. S., Brown M. M., Thompson A. J., Frackowiak R. S. J. (2003). Neural correlates of motor recovery after stroke: a longitudinal fMRI study. *Brain*.

[5] Clarkson A. N., Huang B. S., MacIsaac S. E., Mody I., Carmichael S. T. (2010). Reducing excessive GABA-mediated tonic inhibition promotes functional recovery after stroke. *Nature*.

[6] Hilton B. J., Anenberg E., Harrison T. C., Boyd J. D., Murphy T. H., Tetzlaff W. (2016). Re-establishment of cortical motor output maps and spontaneous functional recovery via spared dorsolaterally projecting corticospinal neurons after dorsal column spinal cord injury in adult mice. *The Journal of Neuroscience*.

[7] Clarkson A. N., López-Valdés H. E., Overman J. J., Charles A. C., Brennan K. C., Carmichael S. T. (2013). Multimodal examination of structural and functional remapping in the mouse photothrombotic stroke model. *Journal of Cerebral Blood Flow & Metabolism*.

[8] Brown C. E., Li P., Boyd J. D., Delaney K. R., Murphy T. H. (2007). Extensive turnover of dendritic spines and vascular remodeling in cortical tissues recovering from stroke. *The Journal of Neuroscience*.

[9] Wahl A.-S., Büchler U., Brändli A. (2017). Optogenetically stimulating intact rat corticospinal tract post-stroke restores motor control through regionalized functional circuit formation. *Nature Communications*.

[10] Dancause N., Nudo R. J. (2011). Shaping plasticity to enhance recovery after injury. *Progress in Brain Research*.

[11] Zeiler S. R., Krakauer J. W. (2013). The interaction between training and plasticity in the poststroke brain. *Current Opinion in Neurology*.

[12] Taub E., Uswatte G., Elbert T. (2002). New treatments in neurorehabiliation founded on basic research. *Nature Reviews Neuroscience*.

[13] Andres R. H., Horie N., Slikker W. (2011). Human neural stem cells enhance structural plasticity and axonal transport in the ischaemic brain. *Brain*.

[14] Brown J. A., Lutsep H. L., Weinand M., Cramer S. C. (2006). Motor cortex stimulation for the enhancement of recovery from stroke: a prospective, multicenter safety study. *Neurosurgery*.

[15] Teskey G. C., Flynn C., Goertzen C. D., Monfils M. H., Young N. A. (2003). Cortical stimulation improves skilled forelimb use following a focal ischemic infarct in the rat. *Neurological Research*.

[16] Lindenberg R., Renga V., Zhu L. L., Nair D., Schlaug G. (2010). Bihemispheric brain stimulation facilitates motor recovery in chronic stroke patients. *Neurology*.

[17] Brown C. E., Aminoltejari K., Erb H., Winship I. R., Murphy T. H. (2009). *In vivo* voltage-sensitive dye imaging in adult mice reveals that somatosensory maps lost to stroke are replaced over weeks by new structural and functional circuits with prolonged modes of activation within both the peri-infarct zone and distant sites. *Journal of Neuroscience*.

[18] Dijkhuizen R. M., Ren J., Mandeville J. B. (2001). Functional magnetic resonance imaging of reorganization in rat brain after stroke. *Proceedings of the National Academy of Sciences of the United States of America*.

[19] Bashir S., Mizrahi I., Weaver K., Fregni F., Pascual-Leone A. (2010). Assessment and modulation of neural plasticity in rehabilitation with transcranial magnetic stimulation. *PM&R*.

[20] Wessel M. J., Zimerman M., Hummel F. C. (2015). Non-invasive brain stimulation: an interventional tool for enhancing behavioral training after stroke. *Frontiers in Human Neuroscience*.

[21] Liew S.-L., Santarnecchi E., Buch E. R., Cohen L. G. (2014). Non-invasive brain stimulation in neurorehabilitation: local and distant effects for motor recovery. *Frontiers in Human Neuroscience*.

[22] Takatsuru Y., Fukumoto D., Yoshitomo M., Nemoto T., Tsukada H., Nabekura J. (2009). Neuronal circuit remodeling in the contralateral cortical hemisphere during functional recovery from cerebral infarction. *The Journal of Neuroscience*.

[23] Iwai M., Stetler R. A., Xing J. (2010). Enhanced oligodendrogenesis and recovery of neurological function by erythropoietin after neonatal hypoxic/ischemic brain injury. *Stroke*.

[24] Zhao Y., Rempe D. A. (2010). Targeting astrocytes for stroke therapy. *Neurotherapeutics*.

[25] Boyden E. S., Zhang F., Bamberg E., Nagel G., Deisseroth K. (2005). Millisecond-timescale, genetically targeted optical control of neural activity. *Nature Neuroscience*.

[26] Li N., Daie K., Svoboda K., Druckmann S. (2016). Robust neuronal dynamics in premotor cortex during motor planning. *Nature*.

[27] Yizhar O., Fenno L. E., Davidson T. J., Mogri M., Deisseroth K. (2011). Optogenetics in neural systems. *Neuron*.

[28] Melchior J. R., Ferris M. J., Stuber G. D., Riddle D. R., Jones S. R. (2015). Optogenetic versus electrical stimulation of dopamine terminals in the nucleus accumbens reveals local modulation of presynaptic release. *Journal of Neurochemistry*.

[29] Nowak D. A., Grefkes C., Ameli M., Fink G. R. (2009). Interhemispheric competition after stroke: brain stimulation to enhance recovery of function of the affected hand. *Neurorehabilitation and Neural Repair*.

[30] Bütefisch C. M., Weβling M., Netz J., Seitz R. J., Hömberg V. (2007). Relationship between interhemispheric inhibition and motor cortex excitability in subacute stroke patients. *Neurorehabilitation and Neural Repair*.

[31] Cheng M. Y., Wang E. H., Woodson W. J. (2014). Optogenetic neuronal stimulation promotes functional recovery after stroke. *Proceedings of the National Academy of Sciences of the United States of America*.

[32] Li S., Overman J. J., Katsman D. (2010). An age-related sprouting transcriptome provides molecular control of axonal sprouting after stroke. *Nature Neuroscience*.

[33] Overman J. J., Clarkson A. N., Wanner I. B. (2012). A role for ephrin-A5 in axonal sprouting, recovery, and activity-dependent plasticity after stroke. *Proceedings of the National Academy of Sciences of the United States of America*.

[34] Carmichael S. T., Kathirvelu B., Schweppe C. A., Nie E. H. (2017). Molecular, cellular and functional events in axonal sprouting after stroke. *Experimental Neurology*.

[35] Dimyan M. A., Cohen L. G. (2010). Contribution of transcranial magnetic stimulation to the understanding of functional recovery mechanisms after stroke. *Neurorehabilitation and Neural Repair*.

[36] Grefkes C., Nowak D. A., Wang L. E., Dafotakis M., Eickhoff S. B., Fink G. R. (2010). Modulating cortical connectivity in stroke patients by rTMS assessed with fMRI and dynamic causal modeling. *NeuroImage*.

[37] Liepert J., Kucinski T., Tüscher O., Pawlas F., Bäumer T., Weiller C. (2004). Motor cortex excitability after cerebellar infarction. *Stroke*.

[38] Machado A. G., Baker K. B., Schuster D., Butler R. S., Rezai A. (2009). Chronic electrical stimulation of the contralesional lateral cerebellar nucleus enhances recovery of motor function after cerebral ischemia in rats. *Brain Research*.

[39] Shah A. M., Ishizaka S., Cheng M. Y. (2017). Optogenetic neuronal stimulation of the lateral cerebellar nucleus promotes persistent functional recovery after stroke. *Scientific Reports*.

[40] Uusisaari M., Knöpfel T. (2010). GlyT2+ neurons in the lateral cerebellar nucleus. *The Cerebellum*.

[41] Uusisaari M., Knöpfel T. (2011). Functional classification of neurons in the mouse lateral cerebellar nuclei. *The Cerebellum*.

[42] Paz J. T., Christian C. A., Parada I., Prince D. A., Huguenard J. R. (2010). Focal cortical infarcts alter intrinsic excitability and synaptic excitation in the reticular thalamic nucleus. *The Journal of Neuroscience*.

[43] Tokuno T., Kataoka K., Asai T. (1992). Functional changes in thalamic relay neurons after focal cerebral infarct: a study of unit recordings from VPL neurons after MCA occlusion in rats. *Journal of Cerebral Blood Flow & Metabolism*.

[44] Iizuka H., Sakatani K., Young W. (1990). Neural damage in the rat thalamus after cortical infarcts. *Stroke*.

[45] Winship I. R., Murphy T. H. (2008). *In vivo* calcium imaging reveals functional rewiring of single somatosensory neurons after stroke. *The Journal of Neuroscience*.

[46] Tennant K. A., Taylor S. L., White E. R., Brown C. E. (2017). Optogenetic rewiring of thalamocortical circuits to restore function in the stroke injured brain. *Nature Communications*.

[47] Starkey M. L., Bleul C., Zörner B. (2012). Back seat driving: hindlimb corticospinal neurons assume forelimb control following ischaemic stroke. *Brain*.

[48] Lindau N. T., Bänninger B. J., Gullo M. (2014). Rewiring of the corticospinal tract in the adult rat after unilateral stroke and anti-Nogo-A therapy. *Brain*.

[49] Brus-Ramer M., Carmel J. B., Chakrabarty S., Martin J. H. (2007). Electrical stimulation of spared corticospinal axons augments connections with ipsilateral spinal motor circuits after injury. *The Journal of Neuroscience*.

[50] Rehme A. K., Eickhoff S. B., Rottschy C., Fink G. R., Grefkes C. (2012). Activation likelihood estimation meta-analysis of motor-related neural activity after stroke. *NeuroImage*.

[51] Wahl A. S., Omlor W., Rubio J. C. (2014). Asynchronous therapy restores motor control by rewiring of the rat corticospinal tract after stroke. *Science*.

[52] Lum P. S., Godfrey S. B., Brokaw E. B., Holley R. J., Nichols D. (2012). Robotic approaches for rehabilitation of hand function after stroke. *American Journal of Physical Medicine & Rehabilitation*.

[53] Mehrholz J., Pohl M., Platz T., Kugler J., Elsner B., Cochrane Stroke Group (2015). Electromechanical and robot‐assisted arm training for improving activities of daily living, arm function, and arm muscle strength after stroke. *Cochrane Database of Systematic Reviews*.

[54] Lu Y., Jiang L., Li W. (2017). Optogenetic inhibition of striatal neuronal activity improves the survival of transplanted neural stem cells and neurological outcomes after ischemic stroke in mice. *Stem Cells International*.

[55] Gage F. H. (2002). Neurogenesis in the adult brain. *The Journal of Neuroscience*.

[56] Alvarez-Buylla A., Seri B., Doetsch F. (2002). Identification of neural stem cells in the adult vertebrate brain. *Brain Research Bulletin*.

[57] Morimoto T., Yasuhara T., Kameda M. (2011). Striatal stimulation nurtures endogenous neurogenesis and angiogenesis in chronic-phase ischemic stroke rats. *Cell Transplantation*.

[58] Song M., Yu S. P., Mohamad O. (2017). Optogenetic stimulation of glutamatergic neuronal activity in the striatum enhances neurogenesis in the subventricular zone of normal and stroke mice. *Neurobiology of Disease*.

[59] Tepper J. M., Tecuapetla F., Koós T., Ibáñez-Sandoval O. (2010). Heterogeneity and diversity of striatal GABAergic interneurons. *Frontiers in Neuroanatomy*.

[60] Young S. Z., Lafourcade C. A., Platel J.-C., Lin T. V., Bordey A. (2014). GABAergic striatal neurons project dendrites and axons into the postnatal subventricular zone leading to calcium activity. *Frontiers in Cellular Neuroscience*.

[61] Gulati T., Won S. J., Ramanathan D. S. (2015). Robust neuroprosthetic control from the stroke perilesional cortex. *The Journal of Neuroscience*.

[62] Bear M. F., Malenka R. C. (1994). Synaptic plasticity: LTP and LTD. *Current Opinion in Neurobiology*.

[63] Buonomano D. V., Merzenich M. M. (1998). Cortical plasticity: from synapses to maps. *Annual Review of Neuroscience*.

[64] Sinnen B. L., Bowen A. B., Forte J. S. (2017). Optogenetic control of synaptic composition and function. *Neuron*.

[65] Gutierrez D. V., Mark M. D., Masseck O. (2011). Optogenetic control of motor coordination by G_i/o_ protein-coupled vertebrate rhodopsin in cerebellar purkinje cells. *The Journal of Biological Chemistry*.

[66] Schröder-Lang S., Schwärzel M., Seifert R. (2007). Fast manipulation of cellular cAMP level by light *in vivo*. *Nature Methods*.

[67] Bérubé N. G., Mangelsdorf M., Jagla M. (2005). The chromatin-remodeling protein ATRX is critical for neuronal survival during corticogenesis. *The Journal of Clinical Investigation*.

[68] Overman J. J., Carmichael S. T. (2013). Plasticity in the injured brain: more than molecules matter. *The Neuroscientist*.

[69] Jan Y. N., Jan L. Y. (2001). Dendrites. *Genes & Development*.

[70] Zahedi A., On V., Ethell I., Bhanu B., Talbot P. (2015). A method to regulate cofilin transport using optogenetics and live video analysis. *Video Bioinformatics*.

[71] Hou S. W., Wang Y. Q., Xu M. (2008). Functional integration of newly generated neurons into striatum after cerebral ischemia in the adult rat brain. *Stroke*.

[72] Ordaz J., Wu W., Xu X.-M. (2017). Optogenetics and its application in neural degeneration and regeneration. *Neural Regeneration Research*.

[73] Carmichael S. T., Saper C., Schlaug G. (2016). Emergent properties of neural repair: elemental biology to therapeutic concepts. *Annals of Neurology*.

[74] Hallett M. (1999). Review: plasticity in the human motor system. *The Neuroscientist*.

[75] Boddington L. J., Reynolds J. N. J. (2017). Targeting interhemispheric inhibition with neuromodulation to enhance stroke rehabilitation. *Brain Stimulation*.

[76] Murase N., Duque J., Mazzocchio R., Cohen L. G. (2004). Influence of interhemispheric interactions on motor function in chronic stroke. *Annals of Neurology*.

[77] Duque J., Hummel F., Celnik P., Murase N., Mazzocchio R., Cohen L. G. (2005). Transcallosal inhibition in chronic subcortical stroke. *NeuroImage*.

[78] Talelli P., Wallace A., Dileone M. (2012). Theta burst stimulation in the rehabilitation of the upper limb: a semirandomized, placebo-controlled trial in chronic stroke patients. *Neurorehabilitation and Neural Repair*.

[79] Kobayashi Z., Akaza M., Endo H., Numasawa Y., Tomimitsu H., Shintani S. (2015). Deterioration of pre-existing hemiparesis due to an ipsilateral internal capsule infarction after a contralateral stroke. *Journal of the Neurological Sciences*.

[80] Seniów J., Bilik M., Lesniak M., Waldowski K., Iwański S., Czlonkowska A. (2012). Transcranial magnetic stimulation combined with physiotherapy in rehabilitation of poststroke hemiparesis: a randomized, double-blind, placebo-controlled study. *Neurorehabilitation and Neural Repair*.

[81] Cheng M. Y., Aswendt M., Steinberg G. K. (2016). Optogenetic approaches to target specific neural circuits in post-stroke recovery. *Neurotherapeutics*.

[82] Lin J. Y. (2011). A user’s guide to channelrhodopsin variants: features, limitations and future developments. *Experimental Physiology*.

[83] Takeuchi N., Izumi S.-I. (2013). Rehabilitation with poststroke motor recovery: a review with a focus on neural plasticity. *Stroke Research and Treatment*.

